# Patella morphological alteration after patella instability in growing rabbits

**DOI:** 10.1186/s13018-017-0615-y

**Published:** 2017-07-11

**Authors:** Jinghui Niu, Qi Qi, Yingzhen Niu, Conglei Dong, Zhenyue Dong, Peng Cui, Fei Wang

**Affiliations:** Third Hospital of Hebei Medical University, No. 139 Ziqiang Road, Shijiazhuang City, Hebei State China

**Keywords:** Knee, Patella, Patella dislocation, Rabbits

## Abstract

**Background:**

The shape of the patella has been considered to be a predisposing factor resulting in patellar instability, but the effects of abnormal patella position during its development are unclear. The present study evaluated patellar morphological changes after patella instability and evaluated the influence of patellar instability on the patella shape.

**Methods:**

Twenty rabbits that were 2 months old were included in the study. The left knee of each rabbit, defined as the experimental group (*N* = 20 knees/group), underwent a medial soft tissue restraint release. The right knee of each rabbit, defined as the control group (*N* = 20 knees/group), did not undergo any surgical procedures. A CT scan was performed on each knee before surgery and 6 months post-surgery to measure the transverse diameter, thickness, Wiberg index, and Wiberg angle for analysis of the patellar morphological changes. Cross-specimen examination was conducted to evaluate the differences between the experimental group and the control group.

**Results:**

The four indices remained the same between the two groups before surgery. However, 6 months after surgery, the mean transverse diameter of the patellae in the experimental group was significantly longer than that in the control group (*P* < 0.001), while the mean thickness in the experimental group was not significantly greater than that in the control group (*P =* 0.314), resulting in a flattened shape. The Wiberg indices were not significantly different between the two groups. However, the mean Wiberg angle was higher in the experimental group than in the control group (*P* < 0.001), which resulted in a flattened articular surface of the patella.

**Conclusion:**

The sectional shape and articular surface of the patella became more flattened after patella instability in this study, which indicates that patella dysplasia could be caused by patella instability. Clinically, early intervention for adolescent patients with patella instability is important.

## Background

The patellofemoral joint is formed by the articulation between the patella and the trochlear groove. The patella is the largest sesamoid bone in the body and sits distal to the muscle bulk of the quadriceps. Geometrically, the patella is shaped like an upside-down triangle [1]. The patella anatomy reveals a median crest traversing in the articular part of the patella, defining a medial and a lateral facet, and the shape-based classification has been proposed by Wibeeg [2]. The patella plays an essential role in knee functions. It acts as a biomechanical lever arm and improves the effective extension capacity of the quadriceps muscle by increasing the moment arm of the patellar tendon. Additionally, it prevents excessive friction between the quadriceps tendon and the femoral condyles [3].

The femoral trochlea consists of the lateral and medial facets of the femoral sulcus [1]. The lateral facet of the femoral trochlea prevents the patella from lateral subluxation and allows it to remain centred in the trochlea during normal knee function [3]. At full knee extension, the patella lies superior to the trochlear cartilage. As the knee flexes to 30°, the patella begins to articulate with the femoral trochlea. Between 30° and 90° of flexion, the inferior part of the patella initially engages with the trochlear, followed by the superior part. Beyond 120° of flexion, the contact area is reduced and only the small odd facet remains in contact with the femur. The contact area is approximately 2.1 cm^2^ at 30° of flexion and increases to approximately 5.5 cm^2^ at 90° of flexion [4, 5]. In addition to the superior and inferior motion of the patella, it also tracks lateral-medial-lateral and tilts laterally during tibiofemoral extension to flexion. The patella translates medially 4 mm when it comes to engage with the trochlear groove and then translates to 7 mm laterally by 90° knee flexion. The patellar medial-lateral rotation is usually less than 3° [6]. Overall, the normal action of the patellofemoral joints is a very complex movement pattern, and the patella comes into contact and is restricted by the femoral trochlea during flexion and extension of the knee.

The relationship between patella instability and trochlear groove morphology has been the topic of extensive research. Trochlear dysplasia has been described as a predisposing factor for patella dislocation [7]. A magnetic resonance imaging (MRI) study demonstrated that patients with instability of the patella exhibit a flatter distal trochlear groove compared to those without the instability [8]. The effect of the position of the patella on the development of the femoral trochlea has been studied and reported. Li et al. [9] and Wang et al. [10] found femoral trochlear dysplasia or flattening after patella instability in growing rabbits. Kaymaz et al. [11] demonstrated that the trochlea flattened after surgery with respect to the patella alta in growing rabbits. These studies indicate that femoral trochlea dysplasia could be caused by instability of the patella.

Although the patella articulates with the femoral trochlea, studies on the correlation between patella morphology and patella instability are lacking. Although patella-shaped disorder is considered as a predisposing factor for patella instability [12], the effect of patella instability on patella morphology development has remained unclear. However, acetabular dysplasia has been proven to be caused by hip dislocation [13, 14]. Considering the similarities between the patellofemoral joint and the hip joint, it may be that patella dysplasia could be caused by patella instability.

Based on the articulation of the patella and femoral trochlea and the similarities between the patellofemoral joint and the hip joint, we hypothesized that early patella instability might lead to morphological alterations in the patella during growth. The objectives of the present study were to elucidate the patellar morphology after instability of the patella in growing rabbits and to discuss the influence of patella instability on patella morphology.

## Methods

### Study design and surgical procedures

The present study was approved by the local Animal Ethics Committee. Forty knees from 20 healthy, 2-month-old New Zealand white rabbits, weighing between 450 and 550 g (provided by the Animal Test Center of the local medical university), were divided into two groups. The left knees comprised the experimental group and were subjected to medial soft tissue restraints release. The right knees formed the control group, and no surgical interventions were performed.

**Fig. 1:**
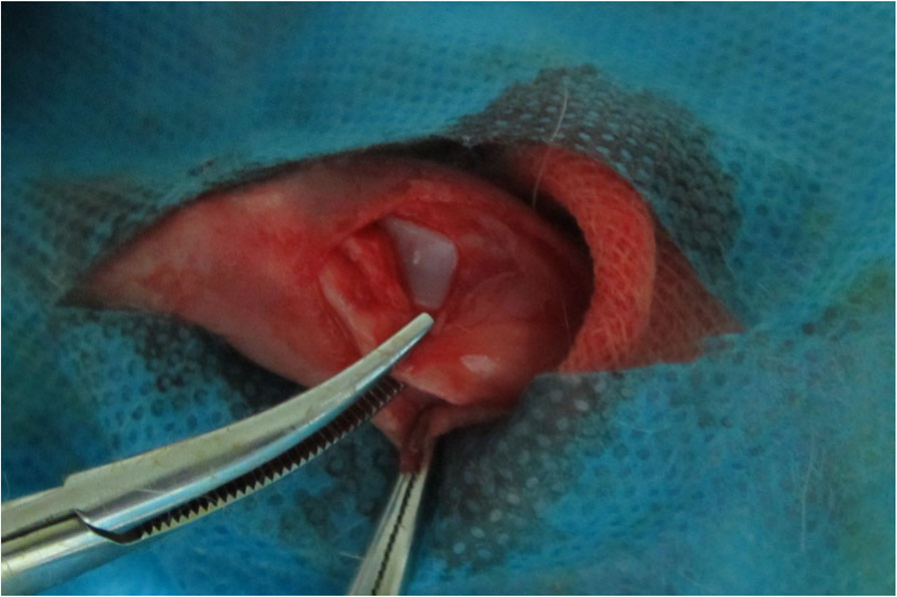
Medial retinaculum and joint capsule was cut

The dislocation procedure was as follows. (1) The rabbits were administered intravenous anaesthesia of ketamine hydrochloride and xylazine at a dosage of 20 and 5 mg/kg body weight, respectively. (2) The knees in the study group were shaved and disinfected by standard procedures. (3) A 3-cm incision was created on the knees in the study group, and the soft tissue was dissected to expose the medial retinaculum and medial side of the joint capsule. (4) A 3-cm longitudinal incision was made along the medial side of the patella to cut the medial retinaculum and joint capsule. Following the release of these structures, patellar instability was seen intraoperatively (Fig. 1). The patella dislocated (the femoral trochlear could be seen) when the knee was flexed, and it returned to the relatively normal position when the knee was extended. (5) After the operation, the incision was sutured, and post-anaesthetic recovery of the rabbit was allowed in an incubator. Caution was exercised to avoid damage to the articular cartilage. Ciprofloxacin (10 mg/kg, po) was administered 3 days after surgery for antibiotic prophylaxis.

The rabbits were raised under the same conditions with water and food. Each animal was housed in an individual stainless steel (310 × 550 × 320 mm) cage, which was sufficiently large for normal activity. According to Masoud et al. [15], skeletal growth and maturation of rabbits is complete at 28 weeks of age. Therefore, all of the rabbits were followed until 6 months post-surgery, after which the animals were sacrificed by venous air embolism.

## Measurements

### CT assessments

**Fig. 2:**
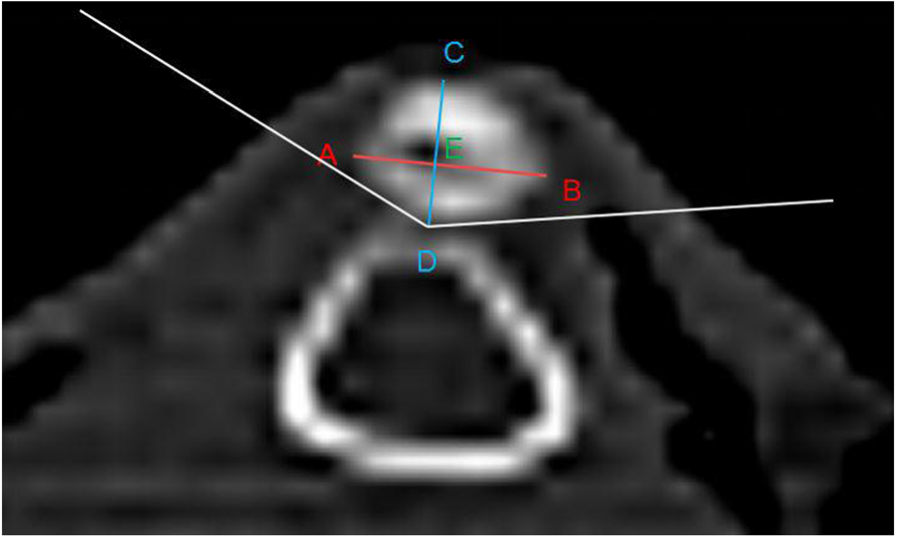
Transverse diameter: length of line AB; thickness: length of line CD; Wiberg index: length of BE/length of AB; Wiberg angle: ∠D

Computerized tomography (CT) scans were performed before surgery and 6 months post-surgery. All rabbits were anaesthetized by the intravenous injection of ketamine hydrochloride and xylazine at a dosage of 20 and 5 mg/kg body weight, respectively, before CT scans were conducted. Six months post-surgery, patella instability was found in every knee in the experimental group after anaesthesia. CT images were captured in the axial plane, which is the optimal position to observe the articulation of the patellofemoral joint. The measurements were analysed by RadiAnt-DICOM software (Medixant Ltd., Poznan, Poland) in the CT machine, which provided an accuracy of 0.1° for angle and 0.01 mm for length (Fig. 2).

The slice image with the widest diameter of the patella was used for the measurements in the transverse plane. Stäubli et al. [16] defined the length between the most medial edge (A) and the most lateral edge (B) of the patella as the transverse diameter (AB). The posterior patellar edge farthest from the baseline was defined as point D. The thickness of the patella was measured by the length of line CD vertical to the baseline. The insertion between line AB and line CD was defined as point E. The Wiberg index (length of BE/length of AB) was calculated, and the Wiberg angle (∠D) was measured as described by Fucentese et al. [17]. All measurements were recorded and averaged by three independent researchers who did not know the grouping.

### Cross observation

CT scans were performed 6 months post-surgery. Subsequently, the rabbits were sacrificed with air venous embolism, and the patellae were dissected and separated. Morphological differences were observed visually.

### Statistics

Statistical analysis was performed using the SPSS version 21.0 (SPSS, IL, USA). Student’s *t* test was used to evaluate the mean difference in the length of AB, the length of CD, and the volume of the Wiberg index and Wiberg angle between the control and experimental groups. A *P* value <0.05 was determined as statistically significant. The results are expressed as mean ± standard deviation.

## Results

### CT assessments

In this study, the differences between the experimental and control groups before surgery were not significantly different (Table 1). However, 6 months post-surgery, the mean transverse diameter (8.20 ± 0.88 mm) of the patella in the experiment group was significantly longer than that in the control group (6.58 ± 0.63 mm) (*P <* 0.001). On the other hand, the mean thickness of the patella in the experimental group (4.83 ± 0.55 mm) was not significantly greater than that in the control group (4.66 ± 0.51 mm) (*P =* 0.314), which resulted in a flattened shape. The Wiberg indices were not found to be significantly different between the two groups. Moreover, the mean Wiberg angle was larger in the experimental group than in the control group (*P <* 0.001), which resulted in a flattened articular surface of the patella (Table 2).

**Table 1:** Measurements before surgery

Measurement	Control group	Experimental group	*P* value
LAB (mm)	3.74 ± 0.42	3.87 ± 0.37	0.303
LCD (mm)	2.73 ± 0.35	2.85 ± 0.30	0.225
Wiberg index	0.50 ± 0.06	0.51 ± 0.05	0.636
Wiberg angle (°)	131.6 ± 6.1	130.5 ± 6.2	0.581

*LAB* length of line AB, *LCD* length of line CD, *Wiberg index* length of BE/length of AB, *Wiberg angle* ∠D

**Table 2:** Measurements at 6 months post-surgery

Measurement	Control group	Experimental group	*P* value
LAB (mm)	6.58 ± 0.63	8.20 ± 0.88	<0.001
LCD (mm)	4.66 ± 0.51	4.83 ± 0.55	0.314
Wiberg index	0.48 ± 0.07	0.51 ± 0.10	0.389
Wiberg angle (°)	131.1 ± 6.2	148.8 ± 11.7	<0.001

*LAB* length of line AB, *LCD* length of line CD, *Wiberg index* length of BE/length of AB, *Wiberg angle* ∠D

### Gross observation

**Fig. 3:**
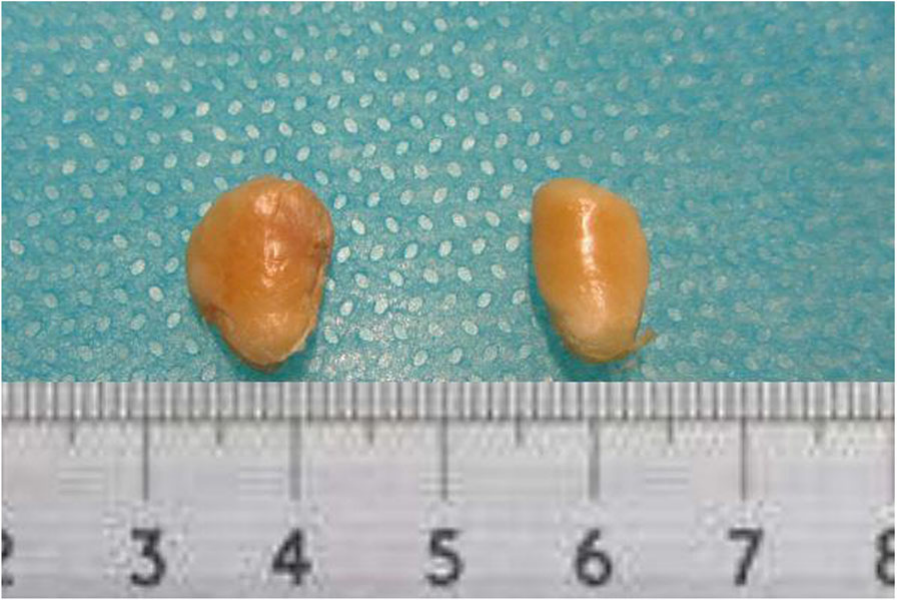
The patella in the experimental group (*left*) is wider than that in the control group (*right*)

**Fig. 4:**
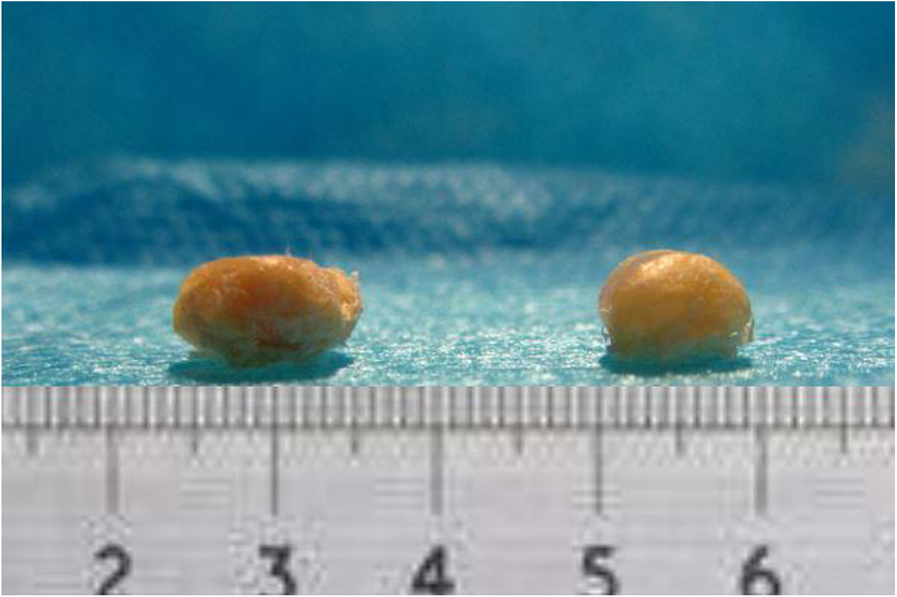
The articular surface in the experimental group (*left*) is more flattened than that in the control group (*right*)

On gross observation, the patella lengths in one rabbit were approximately similar. The patella was wider (Fig. 3), and the articular surface was more flattened (Fig. 4) in the experimental group than in the control group.

## Discussion

The key finding of the present study was that after patella instability in rabbits, the patella becomes more flattened in shape and in the posterior patellar edge than the normal, which may cause problems for patellofemoral stability.

Fucentese et al. [17] studied the patellar morphology in trochlear dysplasia and found a smaller medial facet, higher Wiberg index, and higher prevalence of type II and type III in comparison with the normal patella. Panni et al. [12] retrospectively examined 105 patients (140 knees) with objective patella instability and found a correlation between patellar shape type C and trochlear dysplasia grade 3 and an association between the patella shape and the patella tilt. The risk factors for patella instability have been studied and reported [18–20]. Askenberger et al. [18] characterized the patellofemoral joint morphology through MRI measurements in skeletally immature children with and without a primary lateral patellar dislocation. The incidence of type C patella according to Wiberg in the patellar dislocation group was significantly higher than that in the control group. Yılmaz et al. [19] compared 20 children with acute patellofemoral dislocation with an age-matched healthy control group and found that the mean length and width of the patella in the two groups were significantly different. From the abovementioned studies, patellar morphology was believed to be correlated with patellar stability; however, the effects of patellar instability on patellar development had not been directly observed. In the present study, the change in patellar morphology caused by patella dislocation was proved, emphasizing the importance of patellar stability for the development of the patella.

Ossification of the patella in humans is a 10-year process. The primary ossification core appears in the centre of the patella between 3 and 6 years of age. The process is complete by age 13–16 years in males and earlier in females [21]. On the other hand, the patellofemoral joint plays an essential role in knee function. Trepczynski et al. [22] demonstrated that the force of the patellofemoral joint ranged from <1 body weight during walking to >3 body weights during high flexion activities. Considering the articulation, ossification, and pressure between the patella and femoral trochlea, the alteration in patellar morphology after patellar instability in children and adolescents makes sense.

Gray and Gardner [23] and Doskocil [24] identified that the joint surface morphology of the knee is determined very early in utero. Walmsley [25] revealed that the articular surface of the patella is divided by a vertical ridge into lateral and medial areas during foetal life, and the transverse ridges of the patella on the articular surface do not appear until after birth when the limb is entirely in use and full extension of the knee joint becomes possible. In this study, the patellar morphology was altered significantly after patella instability, which indicates that patella shape is effected by epigenetic factors. Based on all the studies, the development of the patella might be influenced by genetic and epigenetic factors.

Li et al. [9] and Wang et al. [10] found that after patella dislocation or subluxation, the femoral groove leads to an increased groove angle and decreased groove height. Kaymaz et al. [11] studied 32 knees from 16 rabbits that were divided into an experimental group (patellar tendon Z-plasty lengthening for patella alta, 16 knees) and control group (no surgical interventions). The study demonstrated that the mean middle and distal trochlear groove angles in the experimental group were significantly higher and that the mean trochlear depths were significantly lower than those in the control group. The higher trochlear groove angle and lower trochlear depth in the experimental groups in the three studies described above corresponded to the flattened posterior patellar surface in the experimental group in the present study. Thus, the patellofemoral joint development, including the patella and femoral trochlear, could be influenced by patellar stability. Therefore, the findings of the animal studies led us to conclude that congenital and traumatic patellar dislocations during childhood should be treated as early as possible to avoid morphological changes in patellofemoral joints.

The current study has some limitations. First, animal models, rather than human subjects, were used in the study. The anatomy and maturity period of rabbits do not match those of humans. Second, the number of rabbits was small, even though it was sufficient for statistical significance. A larger number of animals would have been optimal for the study.

The present study aimed to investigate the influence of patella instability on the development of the patella. We demonstrated that the sectional shape and articular surface of the patella became more flattened after patella instability in growing rabbits. These observations could be utilized as evidence that patella development is influenced by epigenetic factors. On the other hand, the findings of this study, combined with those of previous studies, show that treatment for patella instability should be conducted early during skeletal development in order to prevent skeletal problems in the future.

## Conclusion

The sectional shape and articular surface of the patella became more flattened after patella instability, which indicates that patella dysplasia could be caused by patella instability. According to the results of this study, it was concluded that treatment for patella instability is better performed early in childhood to prevent patella dysplasia that is likely to be encountered in the future.

## Abbreviations

CT: Computerized tomography
MRI: Magnetic resonance imaging
